# Supplementary material for: Splice-Junction-Based Mapping of Alternative Isoforms in the Human Proteome
Source: Cell Rep. Author manuscript; Available in PMC 2020 Jan 15. (PMC6961840; doi:10.1016/j.celrep.2019.11.026)

A

sp|Q9UMQ6|CAN11_HUMAN|ENSG00000137225|RI1|4898|chr6|44179630|44180163|+2|r13|T4
DPVGVAGHLDGVQARSVGGGTYLQPCPAR q value: 0.0060112 Tr_novel:TRUE RefSeq_Novel:TRUE
Search result spec prec mz: 978.8232 Actual spec prec mz: 978.82323
Fragments matched per AA: 2.14 Proportion of top 20 peaks matched: 0.3

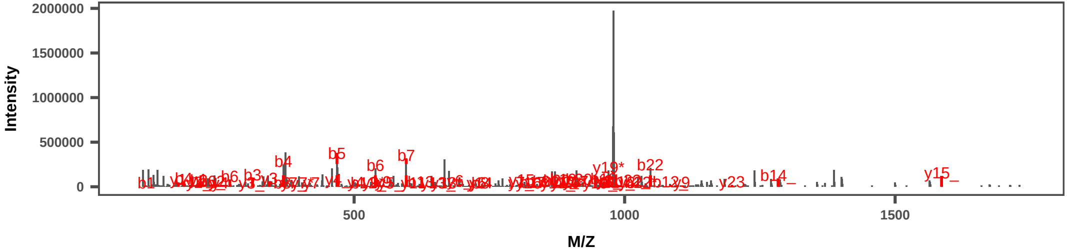

B

Scatterplot of predicted elution time
Fitting R2: 0.757
Novel peptide residual Z score: 1.29
Number of peptides: 3841

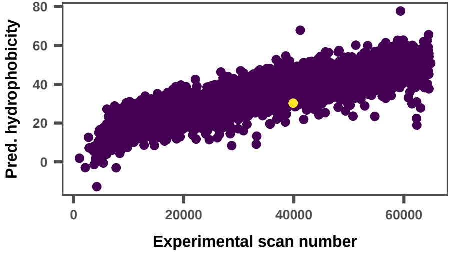

C

Distributions of residuals from best−fit line
of predicted RT vs Expt. scan number
Line: Z score of novel peptide
Z: 1.29

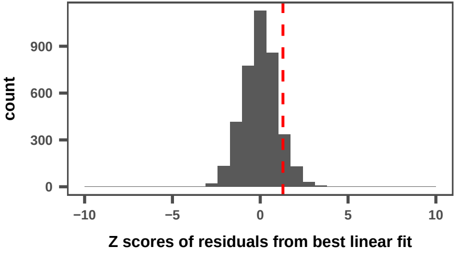

Supplement: 2 [file NIHMS1546469-supplement-2.zip › DF1/PXD009737/TestisTrypsin/TestisTrypsin_66_CAPN11_DPVGVAGHLDGVQARSVGGGTYLQPCPAR.pdf]
